# Systematic Analysis of the Application and Inappropriate Use/Misuse of Statistics in Cholangiocarcinoma Research in Southeast Asia

**DOI:** 10.31557/APJCP.2020.21.2.275

**Published:** 2020

**Affiliations:** 1 *Chulabhorn International College of Medicine, Thammasat University, Paholyothin Road, Klongluang, Pathumthani Thailand, *; 2 *Department of Biostatistics, Christian Medical College, Vellore-632 002, India. *

**Keywords:** Systematic review, statistics, statistical misuse, cholangiocarcinoma

## Abstract

**Objective:**

The aim of the study was to perform a systematic review of research articles related to cholangiocarcinoma (CCA), the bile duct cancer in Southeast Asian (SEA) countries published during 2010-2015 including analysis of inappropriate use/misuse of statistics.

**Methods:**

Research articles were retrieved from the PubMed database using different ‘keywords’ for seven research disciplines/categories in biomedical sciences (medicine/physiology, epidemiology, immunology, pharmacology and toxicology, diagnosis/diagnostics, drug resistance, and biochemistry).

**Results:**

A total of 353 articles were finally included in the analysis based on the pre-defined eligibility criteria. Most were articles of which the studies were conducted in Thailand (335 articles, 94.90%). Disease diagnosis/diagnostics (n=266, 75.35%), biochemistry (n =223, 63.17%), and pharmacology and toxicology (n =218, 61.76%) were the three main research disciplines/categories for CAA conducted in SEA countries during 2010-2015. Thailand was the country which most published CCA-related research articles in all disciplines/categories. Drug resistance was the research category that most applied both descriptive and inferential statistics (100%). The student’s t-test was the most applied test (35.13%). Inappropriate use/misuse of statistics in all types was highest in diagnosis/diagnostics (73.59%) and pharmacology and toxicology (73.06%) research disciplines/categories and was lowest in medicine/pathophysiology (0.26%). Inappropriate use/misuse in almost all types (seven types) was found in the diagnosis/diagnostics category.

**Conclusion:**

Results of the systematic analysis of CCA-related research articles published from the ten SEA countries during 2010-2015 reveal high rates of inappropriate use/misuse of statistics. The readers should be aware of the reliability of the articles and the possibility of wrong interpretation and conclusion of these articles.

## Introduction

Infectious- and non-infectious related diseases remain the major public health problems in the Southeast Asian (SEA) countries (Thailand, the Lao People’s Democratic Republic, Union of Myanmar, Kingdom of Cambodia, Republic of the Philippines, Socialist Republic of Vietnam, Republic of Singapore, Malaysia, Republic of Indonesia, and Brunei Darussalam). Due to similar geographical and contributing factors, the incidence and patterns of these diseases are generally comparable in these countries. Nevertheless, the focus of research priorities on particular research disciplines in these countries may be different depending on specific problems and research facilities, resources, and expertise. Cholangiocarcinoma (CCA), the cancer of bile ducts, is one of the most important infectious-related diseases in SEA. This cancer is one of the most challenging types of cancer due to the lack of a tool for early diagnosis as well as effective chemotherapeutics (Macias, 2014). The significant risk factor for CCA in SEA countries including Thailand is the consumption of improperly cooked cyprinoid fish which contains infective metacercaria of the liver fluke *Opisthorchis viverrini, O. felines*, and *Clonorchis sinensis*, together with dimethylnitrosamine (DMN) from fermented meat (Na-Bangchang and Karbwang, 2014). The highest incidence of CCA worldwide are reported in the northeastern region of Thailand (98.8-317.6 cases/100,000 population/year). For other SEA countries, accurate statistics are not available although a high prevalence of *O. viverrini* infection has been reported in parts of the Lao PDR and Cambodia (Khuntikeo et al., 2018; Chaiteerakij et al., 2017). This has encouraged active CCA-related research on various aspects in SEA countries, either basic, applied, or translational research. Appropriate use of statistical approaches or methods for data analysis is therefore essential for assuring accurate and reliable data interpretation and conclusion (Ercan et al., 2007). 

The aim of the study was to perform a systematic review of research articles related to CCA in SEA countries published during 2010-2015 including analysis of inappropriate/misuse of statistics. The information obtained would provide an accurate perception of the reliability of the results and study conclusions of CCA-related research articles from SEA, and thus, further approaches to avoid/reduce the errors.

## Material and Methods

*Search strategy*

Research articles were retrieved from the PubMed database using the ‘keywords’ for different research disciplines/categories as presented in Table 1 (Khan et al., 2012). No other search conditions were applied. Each research article was classified into at least one of the seven academic disciplines/categories as follows: (i) medicine and pathophysiology; (ii) epidemiology; (iii) immunology and vaccines; (iv) pharmacology and toxicology; (v) diagnosis/diagnostics; (vi) drug resistance; and (vii) and biochemistry.

*Study selection*

The articles identified from the database were initially screened by titles and abstracts to exclude any irrelevant articles. The articles included after the title and abstract screening were further evaluated based on full-texts, applying the predefined eligibility criteria. 

*Eligibility criteria*

Relevant articles were included in the analysis if they met all of the following criteria: (i) articles related to CCA published in PubMed database during January 2010 to December 2015; (ii) articles available as full texts in English; and (iii) studies conducted in one of the ten SEA countries. Articles were excluded if they were: (i) duplicated articles; (ii) review articles including systematic analysis and meta-analysis; or (iii) letters or editorials. All the retrieved articles were carefully monitored to assure their eligibility criteria and stored in EndNote version X7.

*Data extraction and analysis*

Two reviewers extracted data independently and resolved disparity by discussion and suggestion from the third reviewer ( Uman, 2011). The information extracted and collected for analysis included: first author’s name and year of publication, the country where the study was conducted, sample size, and academic discipline/category. Excel spreadsheet (Microsoft, WA, USA) was used for data collection and management. Qualitative data are presented as numbers and percentages, and where appropriate as bar diagrams.

**Figure 1:**
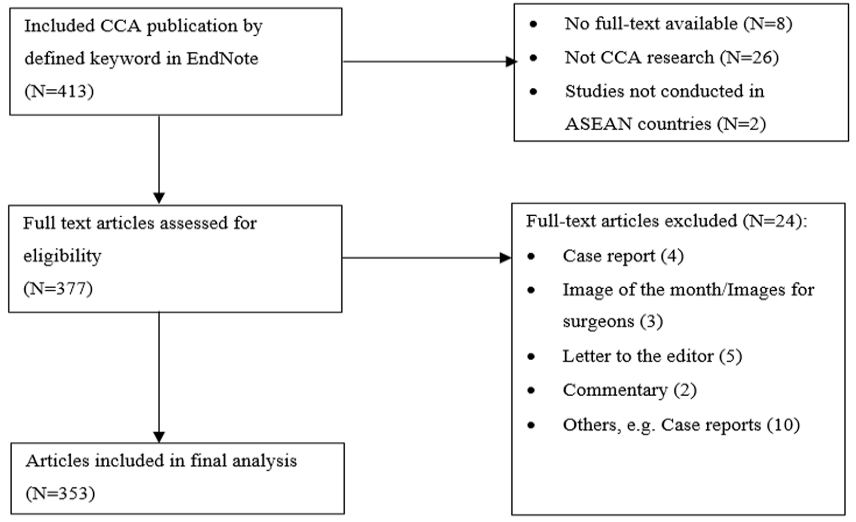
Flow Diagram of Study Identification and Selection

**Table 1:** Keywords Used to Retrieved CCA-Related Research Articles from the ten SEA Countries (source: adapted from Khan et al., 2012).

No	Research discipline/Category	Keyword
1	Medicine and Pathophysiology	Cholangiocarcinoma AND (Clinical trials OR Pathophysiology) Bile duct cancer AND (Clinical trials OR Pathophysiology)
2	Epidemiology	Cholangiocarcinoma AND (Epidemiology) Bile duct cancer AND (Epidemiology)
3	Immunology and Vaccines	Cholangiocarcinoma AND (Immunology OR Vaccines)” Bile duct cancer AND (Immunology OR Vaccines)
4	Pharmacology and Toxicology	Cholangiocarcinoma AND (Pharmacology OR Toxicology OR Anticancer OR Anticancer drug OR Therapeutic OR Therapy) Bile duct cancer AND (Pharmacology OR Toxicology OR Anticancer OR Anticancer drug OR Therapeutic OR Therapy)
5	Diagnosis/diagnostics	Cholangiocarcinoma AND (Diagnostics OR Molecular techniques OR Clinical diagnosis) Bile duct cancer AND (Diagnostics OR Molecular techniques OR Clinical diagnosis)
6	Drug resistance	Cholangiocarcinoma AND (Drug resistance OR Multidrug resistance) Bile duct cancer AND (Drug resistance OR Multidrug resistance)
7	Biochemistry	Cholangiocarcinoma AND (Biology OR Biochemistry OR Parasite biology OR Parasite genetics OR Parasitemia OR Biomarkers) Bile duct cancer (Biology OR Biochemistry OR Parasite biology OR Parasite genetics OR Parasitemia OR Biomarkers)

**Table 2:** Statistical Approaches Applied in the 353 CCA-Related Research Articles Included in the Analysis. Data are presented as numbers of research articles and percentages (%).

Statistical approach applied	n (%)
No statistics was applied used or only percentage was presented	43 (12.18)
Only descriptive statistics was applied	44 (12.46)
Both descriptive and inferential statistics were applied	266 (75.35)

**Figure 2:**
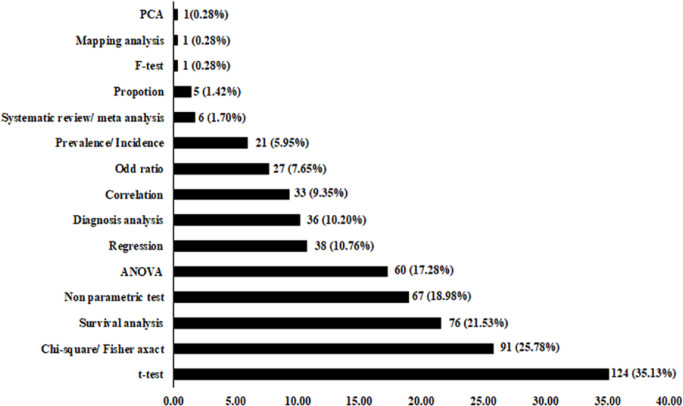
Bar Diagram Summarizing Statistical Analysis Applied in the 353 Included CCA-Related Research Articles

PCA, Principal Component Analysis; ANOVA, Analysis of variance; F-test, test of population variances equality; t-test, student t-test for sample mean

**Table 3:** Statistical Approaches Applied in Each Research Discipline/Category for CCA-related Research Articles Included in the Analysis. Data are Presented as Numbers and Percentages (%) of Total Number of Research Articles in each Discipline/Category

Research	No statistics applied or only percentage was presented	Only descriptive statistics was applied	Both descriptive and inferential statistics were applied
Discipline/Category			
Medicine and Pathophysiology	1 (33.33)	0 (0.00)	2 (66.67)
Epidemiology	6 (7.41)	19 (23.46)	56 (69.14)
Immunology and Vaccines	7 (13.46)	4 (7.69)	41 (78.85)
Pharmacology and Toxicology	27 (14.21)	25 (13.16)	138 (72.63)
Diagnosis/diagnostics	30 (11.90)	26 (10.32)	196 (77.78)
Drug resistance	0 (0.00)	0 (0.00)	16 (100.00)
Biochemistry	26 (12.38)	19 (9.05)	165 (78.57)

**Table 4:** Summary of the Number of Inappropriate Use or Misuse of Statistics CCA-Related Articles in Different Research Disciplines/Categories. *Data are presented as number and percentage (%) of all types of statistic misuse (More than one types of inappropriate use/misuse of statistics were found in some articles).

	Medicine and Pathophysiology	Epidemiology	Immunology and vaccines	Pharmacology and toxicology	Diagnosis/ diagnostics	drug resistance	Biochemistry	Total number of statistical misuse/Total number of articles (%)*
								
No information on sample size estimation procedure provided	1	57	41	138	200	16	166	619/268 (26.26%)
Unclear data distribution	0	36	26	108	129	12	112	423/175 (17.94%)
Unclear statistical hypothesis (1-tailed or 2-tailed)	0	19	24	67	94	6	76	880/120 (37.34%)
Inappropriate data presentation	1	20	17	49	66	7	49	209/90 (8.87)
Lack of information on the statistical significance level (a)	0	8	1	6	10	0	8	33/20 (1.40%)
Application of ANOVA test without information about post-hoc multiple comparison	0	3	12	26	33	3	19	96/42 (4.07)
Use of standard error of mean (SEM, SE) instead of standard deviation (SD) to present data dispersion	0	0	3	31	22	3	18	77/39 (3.27)
Inappropriate use of graphic presentation	0	2	0	4	6	5	3	20/7 (0.85)
TOTAL*	2 (0.26)	145 (19.05)	124 (16.29)	556 (73.06)	560 (73.59)	52 (6.83)	451 (59.26)	2,357/761 (100)

## Results

A total of 413 research articles relating to CCA research were retrieved from the PubMed database. Thirty-six articles were excluded after the screening of the titles and abstracts. Twenty-four full-text articles not fulfilling the eligibility criteria were further excluded. Finally, a total of 353 articles were included in the analysis (Supplement file 1). Of the included articles, most were studies conducted in Thailand (335 articles, 94.90%). A flow chart summarizing the inclusion and exclusion criteria of the searched articles is presented in Figure 1. 

Statistical approaches applied in the 353 articles included in the analysis are summarized in Table 2. Most articles (n=266, 75.35%) applied both descriptive (summarization of the data for central tendency and dispersion) and inferential (hypothesis testing to draw the conclusion to the population using sample data) statistics for data analysis. Disease diagnosis/diagnostics (n=266, 75.35%), biochemistry (n =223, 63.17%), and pharmacology and toxicology (n =218, 61.76%) were the three main research disciplines/categories for CAA research conducted in SEA countries during 2010-2015. Thailand was the country which most published CCA-related research articles in all of the seven disciplines/categories, i.e., medicine and pathophysiology (n=3, 100%), epidemiology (n=72, 70.59%), immunology and vaccines (n=47, 75.81%), pharmacology and toxicology (n=177, 80.45%), diagnosis/diagnostics (n=239, 89.51%), drug resistance (n=16, 100%), and biochemistry (n=205, 91.03%). Table 3 summarizes statistical approaches applied in each research discipline/category for CCA-related research articles included in the analysis. Drug resistance was the research category that most applied both descriptive and inferential statistics (100%). Epidemiology was the research discipline that most applied only descriptive statistics (23.40%). Medicine and pathophysiology were the research discipline which most did not applied statistics (33.33%).

Statistical analysis tests applied for data analysis in the 353 included articles are summarized in Figure 2. Most were parametric tests for data conforming to normal distribution. Student’s t-test was the most applied test (35.13%), followed by chi-square (25.78%), survival analysis (21.53%), non-parametric tests, e.g., Mann-Whitney test, Wilcoxon signed-rank test and Kruskal Wallis test (18.98%), ANOVA (17.28%), regression analysis including linear and logistic regression analysis (10.76%), diagnosis test (sensitivity, specificity, Receiver Operating Characteristic or ROC) (10.20%), correlation analysis including Pearson and Spearman correlation tests (9.35%), odd analysis (7.65%), prevalence/incidence analysis (5.95%), systematic review/meta-analysis (1.70%), proportion (1.41%), F test (0.28%), mapping analysis (0.28%), and principal component analysis of PCA (0.28%). 

Table 4 summarizes the number of inappropriate use/misuse of statistics in CCA-related research articles in different research disciplines/categories. Eight categories of major inappropriate use/misuse of statistical analysis were observed. These included (i) no information on sample size estimation provided, (ii) unclear data distribution (normal or non-normal distribution), (iii) unclear statistical hypothesis (one- or two-tailed), (iv) inappropriate data presentation, (v) lack of information on statistical significance level (α), (vi) application of ANOVA test without information about post-hoc multiple comparison, (vii) use of standard error of mean (SEM) instead of standard deviation (SD) to present data dispersion, and (viii) inappropriate use of graphic presentation. Inappropriate use/misuse of statistics in all types was highest in diagnosis/diagnostics (73.59%) and pharmacology and toxicology (73.06%) research disciplines/categories and was lowest in medicine and pathophysiology (0.26%). Percentages of each type of inappropriate use/misuse vary with research disciplines/categories. In general, inappropriate use/misuse in almost all types (seven types) was found in the diagnosis/diagnostics category; only the misuse of SEM to present data dispersion was found in pharmacology and toxicology discipline.

## Discussion

Statistics is an important tool that assists researchers in obtaining accurate and valid interpretations and conclusions of their valuable research data (Thiese et al., 2015). Unfortunately, inappropriate use/misuse of statistics are commonly found in all research disciplines including biomedical sciences. This systematic review focused on the analysis of the application of statistics in the analysis of CCA-related research articles in different biomedical sciences disciplines/categories from ten SEA countries. PubMed search during 2010-2015 revealed that most (94.90%) of the CCA-related articles in all research disciplines/categories (70.59-100%) were from Thailand. It is also noted that all research articles (100%) in medicine and pathophysiology were published by researchers from Thailand. This supports the statistics showing that Thailand is the country with the highest incidence of CCA in the world (Khuntikeo et al., 2018).

The statistical approaches (descriptive or inferential statistics) and analysis tests (various types of parametric and non-parametric tests) applied in research depends principally on the objective of the studies (descriptive or hypothesis testing), types of data (qualitative or quantitative), and distribution of the tested data (normal or non-normal distribution). The level of complexity of statistical analysis (basic or advanced) may vary according to research types and disciplines (Chernick and Friis, 2003). Inappropriate use/misuse of statistics may therefore also differ with research disciplines/categories depending on the statistics background knowledge and experience of the researchers in a particular discipline. In addition, the competence and experience of the journal’s reviewers on the statistical analysis section is also an important factor that would assist the authors in publishing scientifically sound research articles with proper statistical analysis. Schatz and colleagues performed a systematic review of a total of 406 research articles published in Archives of Clinical Neuropsychology during 1990–1992, 1996–2000, and 2001–2004 (Schatz et al., 2005). Four main types of inappropriate use/misuse of statistics were found. Inappropriate use/misuse of statistics was found highest with (i) negligence of effect size (77.1%), followed by (ii) inappropriate use of P-values (72.2%), (iii) increased chance of Type I error (67.7%), and (iv) inappropriate use of null hypothesis (47.8%). 

In the present systematic analysis, only obvious inappropriate/misuse of statistics was detected since adequate information (raw data, study design, data distribution, and sample size estimation, etc.) for statistical analysis was not provided. As the aim of most research articles was hypothesis testing (e.g., comparison of quantitative or qualitative data between two or more than two groups), most (75.35%) of the research articles applied both inferential (for hypothesis testing) and descriptive (for data summarization) statistics. Drug resistance was the research category that most applied both statistical approaches. The most applied parametric t-test (35.13%) and Chi-square/Fisher exact tests (25.78%) for analysis of quantitative and qualitative data, respectively. Approximately equal proportions of the research articles applied only descriptive (12.46%) or no statistics (12.18%) in the studies. Epidemiology (23.40%) was the research discipline that most applied only descriptive statistics and medicine and pathophysiology (33.33%) was the research discipline that most did not apply statistics at all. Among research articles that applied statistics in data analysis, inappropriate/misuse of statistics was found highest in the diagnosis/diagnostics (73.59%) and pharmacology and toxicology (73.06%) research disciplines. Percentages of each type of inappropriate use/misuse vary with research disciplines/categories. Unclear statistical hypothesis (one- or two-tailed) was the main type of inappropriate use/misuse of statistics (37.34%). In general, inappropriate use/misuse in almost all types (seven types) was found in the diagnosis/diagnostics category; only the misuse of SEM to present data dispersion was found in pharmacology and toxicology discipline. Results of the analysis suggest that several researchers did not aware of the importance of statistical test assumption prerequisites to any statistical test. The commonly used statistical analysis methods from previously published articles with similar study designs are usually followed, which in many cases are incorrect. One of the most common errors in the presentation of mismatched descriptive and interferential statistics (for example, using mean and SD to summarize data with non-normal distribution). Furthermore, most of the currently used statistical software is too simple to use (‘user-friendly’) without the requirement of basic knowledge/experience of researchers on statistics. This results in the high number of articles with inappropriate use/misuse of statistics. 

Improper use/misuse of statistics can distort data interpretation and conclusion and mislead the unwary readers. In addition, it is a waste of time and resources (Thiese et al., 2015). To avoid/reduce errors from inappropriate use/misuse of statistics, clear study protocol and statistical analysis plan should be prepared before the study conduct with consultations of experienced statisticians. The plan should include the aim of study (descriptive or hypothesis testing), selection of appropriate study design, statistical hypothesis (for hypothesis testing studies: one- or two-tailed), sample size estimation (if required), sampling method (if required), types of data (qualitative or quantitative) to be collected, data collection procedure, data analysis approach (data distribution testing, parametric or nonparametric tests), statistical significance level (α), and data presentation, interpretation and conclusion. Statistical test assumptions (e.g., data distribution) must be ensured prior to the application of any statistical test. Sample size estimation and its requirement depend on the nature of the study (descriptive or inferential, quantitative or qualitative, etc.). Although sample size estimation module is available in several statistical softwares (e.g., G*power, OpenEpi, STATA, and n4studies), input of correct required information (e.g., a priori information about parameters of interest, effect size, confidence level, Type I and II errors) requires clear understanding of the nature of the study as well as knowledge on statistics. The limitation of the current study is incomplete coverage of relevant articles included in the study due to language (non-English) or content (non-full text) issues (Figure 1). In addition, no statistical analysis was described in some articles. 

In conclusion, the results of the systematic analysis of CCA-related research articles published from the ten SEA countries during 2010-2015, reveal high rates of inappropriate use/misuse of statistics. The readers should be aware of the reliability of the articles and the possibility of wrong interpretation and conclusion of these articles (Ercan, 2007). The information obtained from the study would provide an accurate perception about the reliability of the results and conclusions reported from CCA-related research articles in SEA, and thus, further approaches to avoid/reduce inappropriate use/misuse of statistics in biomedical sciences.

## Fundings

Ms. Panida Kongjam received financial support from Thammasat University Research Fund under the TU Research Scholar (Contract No.137/2560) and Thammasat University Ph.D. program scholarship.
